# Both dissociations need to be considered: A response to Majerus et al.

**DOI:** 10.1111/jnp.70037

**Published:** 2026-03-10

**Authors:** Tobias Bormann

**Affiliations:** ^1^ Klinik für Neurologie und Neurophysiologie Universitätsklinikum Freiburg, Medizinische Fakultät, Albert‐Ludwigs‐Universität Freiburg Freiburg Germany

There is a debate over the adequate conceptualization of verbal short‐term or working memory, that is, the retention of a limited number of items and their position within a respective sequence. Some researchers view short‐term memory as involving separate stores or buffers that hold information independently of long‐term memory. Others conceive of working memory as activated long‐term memory (henceforth aLTM), supplemented by a mechanism to specifically represent order information (e.g. Majerus, 2019) or maintain items in a highly accessible state (e.g. focus of attention, Cowan, 2019). Colleagues and I reported a double dissociation among two groups of individuals with neuropsychological impairments that we took as support for cognitive models with separate buffers.

Majerus, Cowan and Oberauer put forward a critique of our conclusions to which I shall respond. We would first like to state that we really see the value of their position (e.g. Bormann et al., 2025). We also agree that dissociations in individuals with neuropsychological impairments require great methodological scrutiny. Majerus et al. put forward three arguments against our conclusions, and we shall respond to each of their points below. We believe that each of their arguments has weaknesses. In general, we believe that they focus too much on one aspect of the reported double dissociation and pay too little attention to the opposite pattern of impairments, namely HS's better short‐term retention of verbal information despite her severe impairments at multiple levels of word processing.

Majerus et al.'s first argument is that those accounts which conceive of working memory as aLTM do not predict that each or any language impairment should affect repetition. They note that language processing involved several different levels and representations and not all are involved in repetition. We would like to respond that proponents of working memory as aLTM often refer to correlations between impaired word processing and impaired verbal short‐term memory and view these correlations as evidence for their theoretical position. For example, they refer to studies by Patterson et al. (1994), Majerus et al. (2007) as well as Martin et al. (1996) reporting correlations between single word or semantic processing and retention of verbal information. Majerus et al. do not propose criteria on which neuropsychological studies to consider and which studies to ignore. Crucially, our participant HS exhibited severe impairments at multiple levels of word processing. This included auditory lexical decisions but also spoken word‐to‐picture matching reflecting both a considerable deficit of lexical processing as well as comprehension of a spoken word's meaning. Thus, we demonstrated a severe deficit in HS in mapping auditory input onto semantic representations. If semantic processing is mandatorily involved in short‐term retention tasks as argued by Majerus et al. (see Majerus et al., 2007; Patterson et al., 1994), HS with her severe deficit in accessing semantic information from auditory input should be affected. However, HS also had poor naming, that means, impaired access to output phonological information from semantics. It is, thus, fair to say that HS had multiple deficits in processing spoken words both on the input as well as the output side and it is also fair to say that her deficits were severe. Yet she was better at repeating back strings of words than individuals with better preserved word processing and naming.

The second argument of Majerus et al. is that in their favoured family of cognitive models, impaired verbal short‐term memory does not necessarily predict impaired language processing. They refer to a study by Martin et al. (1996) who ‘lesioned’ a computational model of word repetition. With artificial lesions, the model produced errors in word repetition tasks that resembled the errors of an individual, NC, with deep dysphasia. Crucially, the error patterns changed when model parameters were altered: By severely increasing activation decay, the model produced semantic errors in word repetition. A milder increase of decay altered the error patterns to formal and non‐word errors. A very similar pattern was observed for their participant NC who apparently exhibited some recovery of functions. This went along with a mild increase in short‐term memory capacity. Majerus et al. argue that within the same computational model, qualitatively different patterns of impairments could be simulated.

This reflects an argument often found in the literature, namely, that previous STM patients have not been investigated thoroughly enough to rule out subtle deficits at the single word level (e.g. Allport, 1984; Buchsbaum & D'Esposito, 2019). But, again, our study also reported the opposite dissociation: HS performed better on short‐term memory tasks than the other participants despite her severe impairments at the single word level. Although we consider Martin et al.'s (1996) study extremely interesting and influential, reference does not help much here for two reasons: First, it is unclear whether a pattern like that of HS could be simulated in that model. That means that only one half of our *double* dissociation was successfully simulated. But, as a second objection, even this demonstration remains incomplete. As has been shown repeatedly (e.g. Dell et al., 1997; Foygel & Dell, 2000) fiddling with parameters like activation decay affected the model's ability to produce correct responses in naming tasks. Increasing decay rate in that model leads to naming errors. But our STM patients performed normally in a standard test of naming. That means that the model has not been shown to simulate repetition without also affecting confrontation naming.

Majerus et al.'s third argument is that their models include alternative mechanisms to represent either order information (Majerus, 2019) or items in the focus of attention (e.g. Cowan, 2019), and these mechanisms could be damaged in STM patients. Again, this does not seem to explain HS’ pattern of performance who, under this logic, would be able to represent order or have a preserved focus of attention but would be severely impaired in accessing meaning from auditory input or activating phonological representations from meaning. In addition, we had already alluded to an ongoing study in which we assessed non‐verbal memory span with the Corsi Block‐Tapping test. We expect an amodal deficit of focus of attention or retention of serial order information to affect non‐verbal tasks. The results have not been fully explored but controls had slightly shorter memory spans for non‐verbal material while four patients with impaired verbal short‐term memory had higher non‐verbal spans.

What then would constitute evidence which Majerus et al. would consider convincing? They make a suggestion: For them, the same items should be used for word processing and repetition tasks. They make this point twice in their commentary. But this suggestion misses the point for two reasons. First, this questions the validity of aphasia tests. We used established tests to evaluate auditory lexical decision, word comprehension and naming. These tests use short, common concrete nouns as items. We used comparable words for repetition tasks (i.e. one‐syllable concrete nouns controlled for frequency). While we did not use exactly the same pool of items, nothing in the sets of words was unusual or idiosyncratic. In contrast, they constitute sets of rather common and easy to process stimuli.

Second, the observation of consistently impaired representations of specific concepts was made almost exclusively in the context of semantic dementia, as acknowledged by Majerus et al. It has been argued that individuals affected by this neurodegenerative disease suffer from specific semantic representations (or, semantic hubs) deteriorating (sometimes also referred to as a storage deficit in contrast to an access deficit, cf. Jefferies & Lambon Ralph, 2006). When assessed repeatedly, some items would be consistently inaccessible while others would consistently be accessible. In contrast, aphasia due to stroke has been conceived of as an access deficit with inconsistent accessibility over repeated assessments (Jefferies & Lambon Ralph, 2006). This means that the suggested studies can only be done with individuals suffering from semantic dementia but that they cannot be done with individuals suffering from stroke. But stroke has been the main cause for impaired short‐term memory. Majerus et al.'s call for methodological scrutiny can only be applied to a very limited group of patients.

To summarize, we greatly appreciate Majerus et al.'s challenge, we see a lot of value in their research and their proposed models, and we certainly agree that more and extremely carefully documented dissociations are needed to settle the issue of word processing in verbal short‐term memory. On the other hand, we do not consider their arguments entirely convincing. In our view, they pay too little attention to the fact that we documented the opposite dissociation with our participant HS. Therefore, arguably, the reported *double* dissociation remains the strongest evidence so far for independent verbal long‐term and short‐term memory.

## AUTHOR CONTRIBUTIONS

**Tobias Bormann:** Conceptualization; writing – original draft; writing – review and editing.

## Data Availability

Data sharing is not applicable to this article as no datasets were generated or analysed during the current study.

## References

[jnp70037-bib-0001] Allport, D. A. (1984). Auditory‐verbal short‐term memory and conduction aphasia. In H. Bouma & D. G. Bouwhuis (Eds.), Attention and performance X (pp. 313–325). Erlbaum.

[jnp70037-bib-0002] Bormann, T. , Hubrich, K. , Otazaghine, L. , & Weiller, C. (2025). Retention of acoustic, phonological, and semantic information in a person with conduction aphasia: Implications for language‐based models of short‐term memory. Aphasiology, 1–15. 10.1080/02687038.2025.2531416

[jnp70037-bib-0003] Buchsbaum, B. R. , & D'Esposito, M. (2019). A sensorimotor view of verbal working memory. Cortex, 112, 134–148. 30639088 10.1016/j.cortex.2018.11.010

[jnp70037-bib-0004] Cowan, N. (2019). Short‐term memory based on activated long‐term memory: A review in response to Norris (2017). Psychological Bulletin, 145, 822–847. 31328941 10.1037/bul0000199 PMC6650160

[jnp70037-bib-0005] Dell, G. S. , Schwartz, M. F. , Martin, N. , Saffran, E. M. , & Gagnon, D. A. (1997). Lexical access in aphasic and nonaphasic speakers. Psychological Review, 104(4), 801–838. 9337631 10.1037/0033-295x.104.4.801

[jnp70037-bib-0006] Foygel, D. , & Dell, G. S. (2000). Models of impaired lexical access in speech production. Journal of Memory and Language, 43(2), 182–216.

[jnp70037-bib-0007] Jefferies, E. , & Lambon Ralph, M. A. (2006). Semantic impairment in stroke aphasia versus semantic dementia: A case‐series comparison. Brain, 129(8), 2132–2147. 10.1093/brain/awl153 16815878

[jnp70037-bib-0008] Majerus, S. (2019). Verbal working memory and the phonological buffer: The question of serial order. Cortex, 112, 122–133. 29887208 10.1016/j.cortex.2018.04.016

[jnp70037-bib-0009] Majerus, S. , Norris, D. , & Patterson, K. (2007). What does a patient with semantic dementia remember in verbal short‐term memory? Order and sound but not words. Cognitive Neuropsychology, 24(2), 131–151. 18416485 10.1080/02643290600989376

[jnp70037-bib-0010] Martin, N. , Saffran, E. M. , & Dell, G. S. (1996). Recovery in deep dysphasia: Evidence for a relation between auditory verbal‐STM capacity and lexical errors in repetition. Brain and Language, 52, 83–113. 8741977 10.1006/brln.1996.0005

[jnp70037-bib-0011] Patterson, K. , Graham, N. , & Hodges, J. R. (1994). The impact of semantic memory loss on phonological representations. Journal of Cognitive Neuroscience, 6(1), 57–69. 23962330 10.1162/jocn.1994.6.1.57

